# Polyphosphoramidate Glycohydrogels with Biorecognition Properties and Potential Antibacterial Activity

**DOI:** 10.3390/molecules30153140

**Published:** 2025-07-26

**Authors:** Zornica Todorova, Oyundari Tumurbaatar, Violeta Mitova, Neli Koseva, Iva Ugrinova, Penka Petrova, Kolio Troev

**Affiliations:** 1 Institute of Polymers, Bulgarian Academy of Sciences, 1113 Sofia, Bulgaria or zornica.todorova@orgchm.bas.bg (Z.T.); oyundari.tumurbaatar@orgchm.bas.bg (O.T.); mitova@polymer.bas.bg (V.M.); koseva@polymer.bas.bg (N.K.); 2 Institute of Organic Chemistry with Centre of Phytochemistry, Bulgarian Academy of Sciences, 1113 Sofia, Bulgaria; 3 Institute of Molecular Biology, Bulgarian Academy of Sciences, 1113 Sofia, Bulgaria; imb@bio21.bas.bg; 4 The Stephan Angeloff Institute of Microbiology, Bulgarian Academy of Sciences, 1113 Sofia, Bulgaria; pepipetrova@yahoo.com

**Keywords:** polyphosphoramidate glycohydrogels, hexamethylene diisocyanate, Concanavalin A, silver nanoparticles, degradation, cytotoxicity, cell adhesion

## Abstract

In the present study, for the first time, a biodegradable and non-toxic polyphosphoramidate glycohydrogel (PPAGHGel) was prepared by crosslinking a polyphosphoramidate glycoconjugate (PPAG) with hexamethylene diisocyanate (HMDI) under mild conditions. Poly(oxyethylene H-phosphonate) (POEHP) was used as a precursor and was converted into PPAG via the Staudinger reaction with glucose-containing azide (2-p-azidobenzamide-2-deoxy-1,3,4,6-tetra-O-trimethylsilyl-α-D-glucopyranose). Then, crosslinking of PPAG was performed to yield PPAGHGel, which was thoroughly characterized. The gel showed a gel fraction of 83%, a swelling degree of 1426 ± 98%, and G″ = 1560 ± 65 Pa. The gel was fully degraded by alkaline phosphatase (400 U/L, pH 9) in 19 days, while hydrolytically, up to 52% degradation was observed under similar conditions. Multivalent studies of the obtained hydrogel with lectin–Concanavalin A were performed. PPAGHGel binds 92% of Concanavalin A within 24 h and the complex remains stable until the amount of glucose reaches 0.3 mM. PPAGHGel acts as a stabilizer for silver nanoparticles (12 nm). SEM shows pores measuring 10 µm (surface) and 0.1 mm (interior) with capillary channels, confirming the gel’s suitability for biosensors, drug delivery, or wound dressings. The cytotoxic (IC50) and cell-adhesive properties of the obtained hydrogel were investigated on human cell lines (HeLa). Antibacterial activity tests were also performed with gel containing silver nanoparticles against skin-associated pathogenic bacteria. The results show that PPAGHGel possesses excellent biocompatibility, non-adhesive properties and antibacterial activity.

## 1. Introduction

By definition, hydrogels are water-swellable, crosslinked, three-dimensional polymer-derived materials. Depending on the method of crosslinking, hydrogels can be classified mainly into three categories: chemical, physical, and biological. When hydrogels contain carbohydrate structures, they are called glycohydrogels, which can contain a large amount of water while maintaining their mechanical strength. Glycohydrogels can be classified based on different criteria: by the method of crosslinking used, by the mechanical characteristics, and by the composition of the hydrogel backbone [1].

Two primary types of polymers are primarily used to produce glycohydrogels: natural polymers and synthetic polymers [2]. Natural polymers are polysaccharides (chitosan, hyaluronic acid, alginate), proteins (gelatin, heparin), and others. Unlike hydrogels based on natural polymers, those based on synthetic polymers allow for control over important properties such as hydrophilic–hydrophobic balance, mechanical strength, and microstructure. On the other hand, most hydrogels based on synthetic polymers do not offer the opportunity for widespread application in biomedicine due to the lack of biological signals for cell proliferation and tissue regeneration. Therefore, modern biomaterials research combines the advantages of natural and synthetic hydrogels. For example, glycohydrogels based on synthetic polymers and also containing multiple carbohydrate units in their structure can possess multivalent properties, can possess bacterial adhesive properties, and can be used as a stabilizer for the preparation of silver metal nanoparticles. Further, incorporating a hydrolytically labile linkage in the precursor main chain allows hydrogels to adopt biodegradable properties.

In the current study, a glycohydrogel based on polyphosphoramidate glycoconjugates (PPAGs) crosslinked with hexamethylene diisocyanate (HMDI) was obtained for the first time. The newly synthesized gel is biodegradable, hydrophilic, non-toxic, and capable of complexing proteins. It also acts as a stabilizer for the preparation of silver nanoparticles.

## 2. Results and Discussion

### 2.1. Synthesis of Poly(oxyethylene H-phosphonate) (POEHP)

The first step in the preparation of the glycohydrogel is the synthesis of poly(oxyethylene H-phosphonate). It is synthesized by a polycondensation reaction between polyethylene glycol with a molecular weight of 600 g/mol and dimethyl H-phosphonate. (See Supplementary Materials).

**Figure 1:**
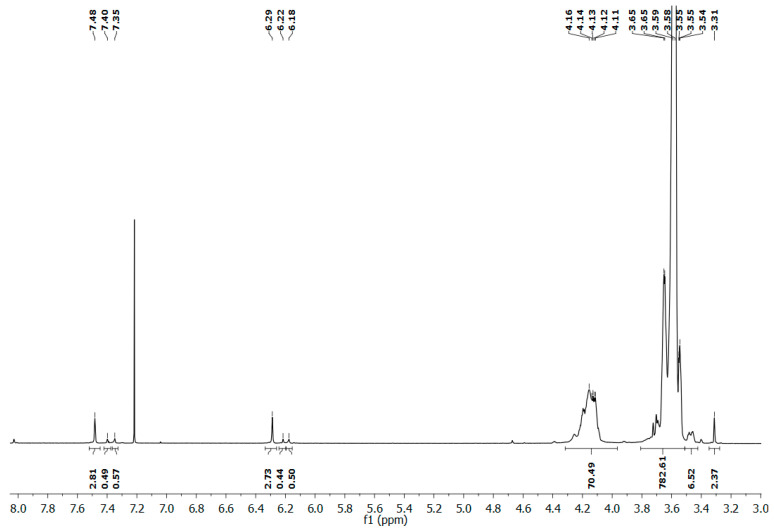
^1^H NMR spectrum of POEHP.

In the ^1^H NMR spectrum of the obtained poly(oxyethylene H-phosphonate) (Figure 1), there are three types of P-H protons, which appear as doublets at 6.89 ppm with ^1^J(P,H) = 717.96 Hz that can be assigned to the P-H protons in the repeating units (–OCH_2_CH_2_OP(O)(*H*)OCH_2_CH_2_O–), at 6.81 ppm with ^1^J(P,H = 711.55 Hz that can be assigned to the P-H protons in the CH_3_O–P(O)(*H*)OCH_2_CH_2_O– end group and at 6.76 ppm with ^1^J(P,H = 704.55 Hz that can be assigned to the P–H protons in the HO−P(O)(*H*)OCH_2_CH_2_O end group.

The multiplet at 4.11–4.16 ppm is assigned to the methylene protons of the phosphoester segments (–CH_2_O(H)P(O)OCH_2_–), and the multiplet at 3.31–3.65 ppm is assigned to the oxyethylene units of the polyether chains.

**Figure 2:**
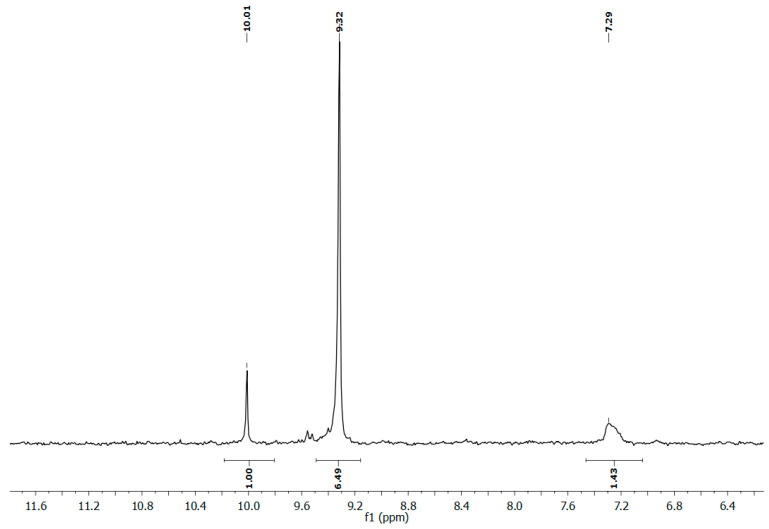
^31^P {H} NMR spectrum of POEHP.

The ^31^P{H} NMR spectrum (Figure 2) of POEHP shows a signal at 9.32 ppm, which corresponds to the P-atoms in the repeating units (–OCH_2_CH_2_O–*P*(O)(H)OCH_2_CH_2_O–), a signal at 10.01 ppm, corresponding to the P-atom in the end group CH_3_O–*P*(O)(H)OCH_2_CH_2_O–, and a signal at 7.29 ppm for the P-atom in the other end group, HO–*P*(O)(H)OCH_2_CH_2_O.

### 2.2. Synthesis of Poly(phosphoramidate) Glycoconjugate via Staudinger Reaction

To prepare the poly(phosphoramidate) glycoconjugate, POEHP and glucose-containing azide (2-p-azidobenzamide-2-deoxy-1,3,4,6-tetra-O-trimethylsilyl-α-D-glucopyranose) were reacted via the Staudinger reaction. The Staudinger reaction is a reaction between a trivalent phosphorus compound and an azide. The first step for preparing poly(phosphoramidate) glycoconjugate is the conversion of the pentavalent phosphorus atom of POEHP into a trivalent one using a silylating reagent—N,O-bis-trimethylsilyl acetamide (BSA). The reaction proceeds at a four-fold molar excess of BSA and in an inert atmosphere. The next step is the Staudinger reaction between the three-coordinate POEHP and the previously synthesized azidosugar, the hydroxyl groups of which should have been previously silylated/protected with the help of another silylating reagent, hexamethyldisilazane (HMDS). The protection of the OH groups of the sugar is an important step, as they are reactive and can lead to read-out side reactions. (See Supplementary Materials).

Another goal in the synthesis of the azidosugar is to directly attach the azide N_3_-group to an aromatic moiety, i.e., to use an aromatic azidosugar in the reaction. This is important, as it has been shown that aromatic azides react with a higher degree of conversion (over 80%) in the Staudinger reaction, compared to aliphatic azides (40–50%) [3]. Upon the attachment of the azidosugars to the phosphorus atoms of POEHP, phosphoramidate bonds are formed. The final step is the desilylation of the poly(phosphoramidate) glycoconjugate and the hydroxyl groups of the sugars with the help of a strong base, tetrabutylammonium fluoride (TBAF), producing the desired final product. (See Supplementary Materials).

**Figure 3:**
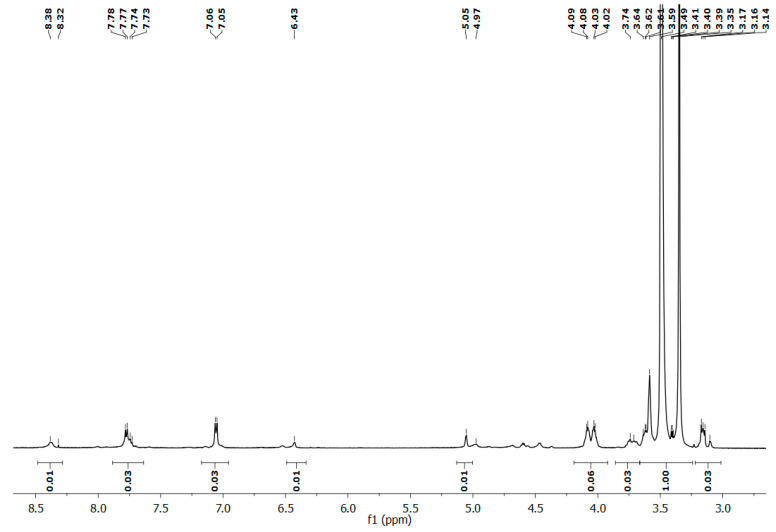
^1^H NMR spectrum of PPAG.

In the ^1^H NMR spectrum (Figure 3) of PPAG, doublets at 7.41 ppm are observed, which are related to the aromatic protons of the azidosugars. These signals prove that attachment of the azidosugars to POEHP has occurred. The poly(phosphoramidate) glycoconjugate was purified by dialysis in water at 0 °C and a neutral pH, in order to avoid hydrolysis of the phosphoroester (P-O-C) bonds in the main chain and the phosphoramidate bond (P-N). Hydrolysis in the system is an undesirable process, as it leads to a deterioration of the molecular mass characteristics of PPAG.

**Figure 4:**
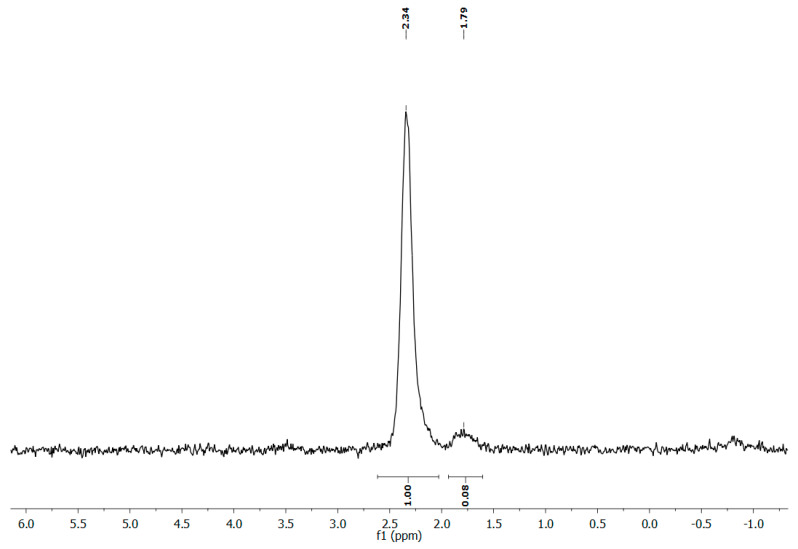
^31^P{H} NMR spectrum of PPAG.

In the ^31^P{H} spectrum (Figure 4), two new signals for the P-atoms appear at 2.34 ppm and 1.79 ppm. The signal at 2.34 ppm refers to the phosphorus atom bonded to the nitrogen atom of the amido group of the repeating unit. The signal at 1.79 ppm refers to the phosphorus atom bonded to the nitrogen atom of the amido group in the terminal P(O)NH(OCH_3_) group. The reaction proceeded to completion quantitatively. The average molecular masses (M_n_) of POEHP and PPAG, as determined by the NMR spectra, are 4800 g/mol and 7200 g/mol, respectively.

### 2.3. Synthesis and Characterization of Polyphosphoramidate Glycohydrogel (PPAGHGel)

**Scheme 1:**
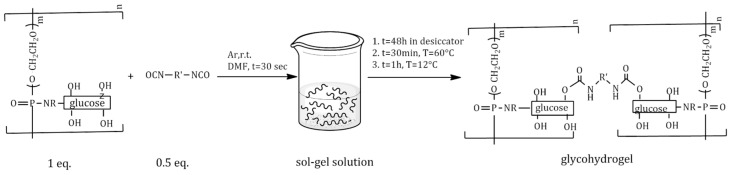
Obtention of poly(phosphoramidate) glycocohydrogel PPAGHGel.

Polyphosphoramidate glycohydrogel (PPAGHGel) was prepared by crosslinking PPAG using hexamethylene diisocyanate (HMDI) as a crosslinking agent; the latter’s isocyanate groups react with the hydroxyl groups of the glucose residues in the PPAG structure under mild reaction conditions (Scheme 1). HMDI is an effective crosslinker for carbohydrate-containing polymers in the synthesis of glycohydrogels due to its chemical reactivity, structural properties, and compatibility with polysaccharide systems. As an aliphatic diisocyanate, HDI leads to the formation of non-yellowing and biocompatible networks. This is particularly advantageous for biomedical applications where color stability and compatibility with biological systems are crucial. HDMI’s reactivity is not limited to a specific type of polysaccharide. It has been successfully used to crosslink a variety of carbohydrate polymers, demonstrating its versatility in forming glycohydrogels with diverse properties [4]. The degree of crosslinking achieved with HMDI can be controlled by adjusting the molar ratio of HMDI:OH-groups and the reaction conditions, allowing for the customization of hydrogel properties such as porosity, mechanical strength, and degradation rate. This tunability is essential for designing glycohydrogels for specific applications. In our study, polyphosphoramidate glycohydrogels were prepared by crosslinking of one hydroxyl group per PPAG polymer unit. The other OH-groups remained free for further multivalent studies or for the stabilization of silver nanoparticles. The gel was prepared in DMF as a common solvent for the crosslinking agent and PPAG.

#### 2.3.1. Swelling and Gel Fraction

The degree of swelling of a hydrogel is determined by the interaction between the polymer chains and the solvent. Hydrogels can be classified according to the water content as follows: low- [5] (%w < 5), medium-, and high-swelling, and superabsorbent (%w > 400) [6] hydrogels. To determine the degree of swelling, the weight of the dry gels was measured first after lyophilization, m_j_, and then after soaking in water/ethanol (80/20%vol) until a constant m_i_ value was reached. Three measurements were performed with an average swelling rate after 1 h of 600% ± 59% and after 15 days of 1426% ± 98%, with a calculated gel fraction of 83.14%. This result proves that the obtained PPAGHGel is a superabsorbent hydrogel.

#### 2.3.2. Mechanical Properties

One of the important characteristics of hydrogels is their mechanical properties, which are determined by the modulus of elasticity, E’ (Young’s modulus), and the loss modulus, G″. G′ and G″ values of the polyphosphoramidate glycohydrogel prepared under dynamic rheological conditions were measured as described in the Materials and Methods section.

**Figure 5:**
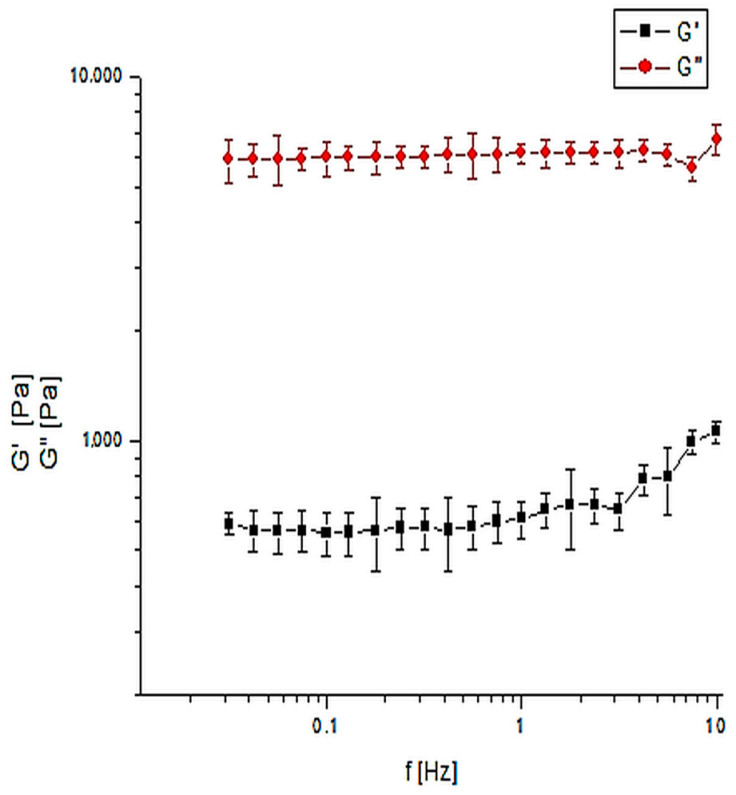
Elastic modulus, G′, and loss modulus, G″, of PPAGHGel.

Figure 5 presents the results of the mechanical properties of the polyphosphoramidate glycohydrogel: elastic modulus: G′ = 5740 ± 74 Pa; loss modulus: G″ = 1560 ± 65 Pa. As can be seen from the figures, G′ is a higher value than G″, i.e., the applied oscillatory force is smaller than the intramolecular forces in the gel.

The hydrogel (PPAGHGel) behaves as an elastic material and is sufficiently strong. In addition, PPAGHGel has the potential to increase its mechanical strength with an increase in the percentage of crosslinking agent. The amount of crosslinking agent strongly impacts the mechanical strength of the hydrogel. Van Dijk et al. have observed that as the percentage of the crosslinking agent increases, the mechanical strength of the hydrogel also increases [7]. The cited article reports on the synthesis of glycohydrogels with similar characteristics as suitable candidates for biomedical applications (wound dressings and drug carriers). Pourjavadi et al. also proposed glycohydrogels that are both strong and flexible for use in wound dressings [8].

#### 2.3.3. Morphology of PPAGHGel

**Figure 6:**
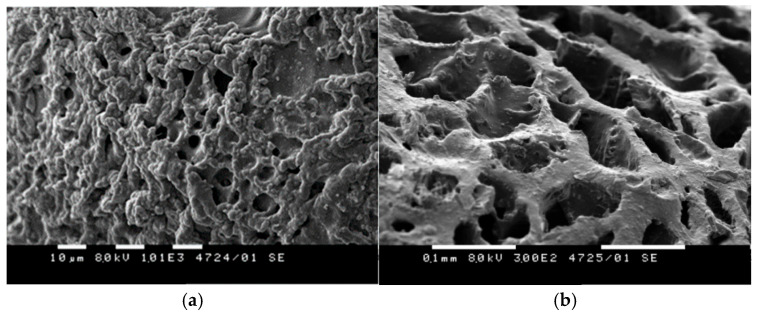
SEM images of PPAGHGel of the (**a**) surface; (**b**) cut.

The structure of the hydrogels, as seen in the SEM images (Figure 6), exhibits channel-like, interconnected pores that can absorb water into the network through capillary action. This morphological characteristic can explain the physical properties, such as hydrophilicity, swelling degree, and strength. The lyophilized hydrogel can be more easily morphologically analyzed than the already swollen gel, since in a swollen state, the gel cannot be metallized. On the other hand, after the lyophilization of the gel, the pores on the surface stick to each other, forming a layer that interferes with the analysis. This is taken into account when reporting the results of the experiments performed with lyophilized samples [9].

The morphology of PPAGHGel was studied by scanning electron microscopy. The SEM images below were taken at random locations on the cross-section of the hydrogels and the pore size value was calculated arithmetically for each sample. PPAGHGel showed a not very homogeneous structure on its surface (Figure 6). This can be explained by the fact that, during lyophilization, the pores adhere to each other on the surface of the gel, forming capillary channels. These pores have an average size of 10 µm. The presence of capillary channels allows water to more easily access the interior of the gel. Inside the gel slice, the measured pore size is 0.1 mm. This morphology of PPAGHGel, consisting of capillary channels and macropores, allows the gels to absorb water in amounts up to 1426 ± 98%. This effect is observed in superabsorbent glycohydrogels [9].

#### 2.3.4. Thermogravimetric Analysis of PPAGHGel

**Figure 7:**
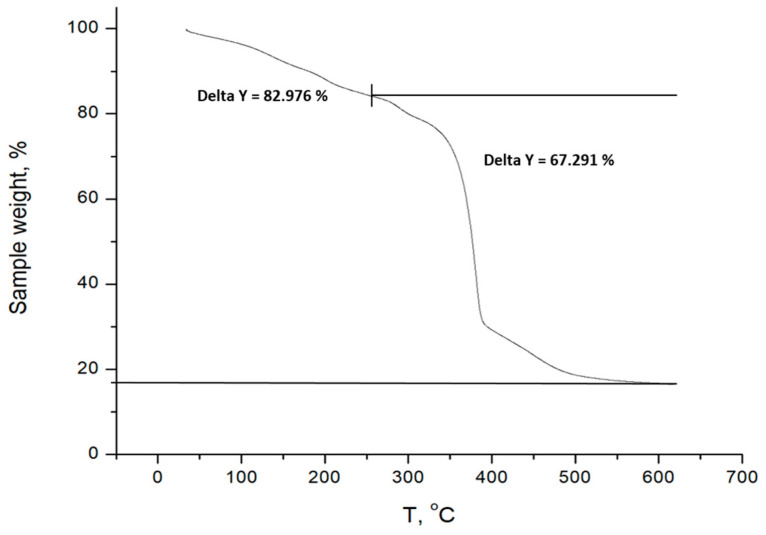
Thermogravimetric analysis of the obtained PPAGHGel.

Thermogravimetric analysis allowed us to determine the thermal stability of the obtained PPAGHGel. The analysis revealed two distinct stages within the temperature range up to 380 °C. The first stage involves the loss of bound water from the gel, while the second stage, corresponds to the dehydration of glucose units. The TGA curve (Figure 7) shows that at 380 °C, PPAGHGel loses 83% of its weight. These findings suggest that the gel can undergo thermal treatment, allowing for the preparation of sterile samples suitable for biomedical applications.

### 2.4. Enzymatic and Hydrolytic Degradation of PPAGHGel

As mentioned in the introduction section, chemical glycohydrogels can be degraded hydrolytically or enzymatically. Enzymatic degradation of polymeric biomaterials is a heterogeneous process influenced by the manner in which enzymes interact with polymer chains. When proteins and polysaccharides are included in their structure, they become enzymatically degradable. Typically, polymer chains are degraded in the following way: (1) the enzyme from the solution reaches the surface of the material; (2) the enzyme is absorbed into the substrate, i.e., forms the enzyme–substrate complex; (3) the enzyme catalyzes the hydrolysis reaction; (4) the final process is the breakdown of the polymer chains into solution.

In this work, the enzyme alkaline phosphatase (ALP) with a molecular mass of M = 86 kDa was used. ALP can be found in all tissues, but it is found in the largest quantities in the liver, bones, cells that cover the bile ducts, kidneys, the mucous membrane covering the intestines, the placenta, and other tissues. ALP exhibits the greatest activity at pH values within the range of the environment, from 8.6 to 10.1. The reference limits of the ALP enzyme in adults are up to 120 U/L; in women, they should be below 105 U/L; in children up to 3 years of age, they should be below 400 U/L; and in people between 12 and 18 years of age, they should be below 380 U/L. To determine the enzymatic degradation of PPAGHG, the absorbance (at λ = 600 nm) of a solution of the enzyme with a concentration of 400 U/L in TRIS-HCl buffer at pH = 9 in which the PPAGHGel was immersed was periodically measured. The results showed that the gel completely disintegrated in 19 days. The same experiment was performed in TRIS-HCl buffer at pH = 9 to determine the hydrolytic degradation of the gel. For the same period, the degree of hydrolytic degradation of PPAGHGel was 52%.

**Figure 8:**
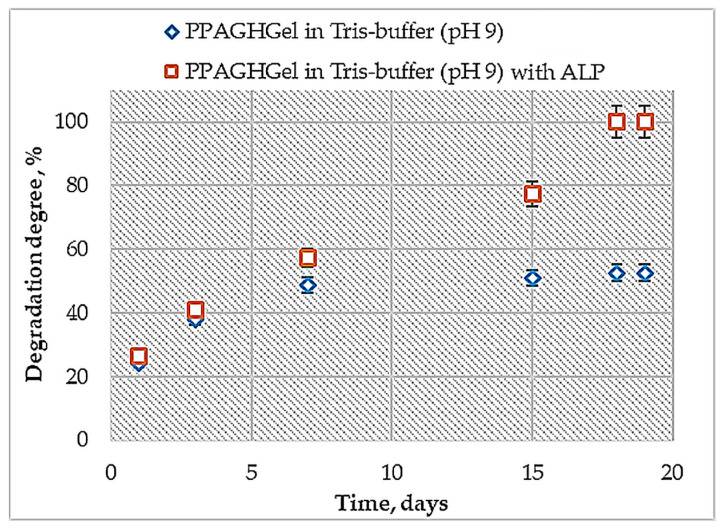
Enzymatic and hydrolytic degradation of PPAGHGel.

**Figure 9:**
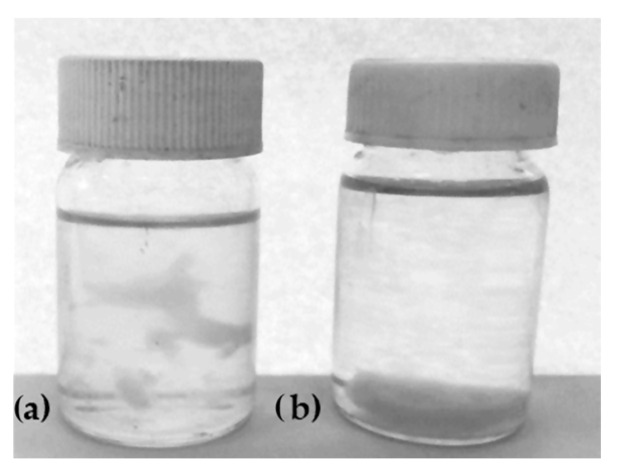
Enzymatic (**a**) and hydrolytic (**b**) degradation of PPAGHGel after soaking for 15 days.

Figure 8 shows the degree of enzymatic and hydrolytic degradation of the PPAGHGel after 7 days: 58% and 42%, respectively. From the 15th day onward, a significant difference in the degree of degradation was observed: enzymatic degradation occurred more quickly than hydrolytic degradation (Figure 9). The quicker enzymatic decomposition of PPAGHGel observed at pH 9 with ALP, compared to the hydrolytic decomposition of it in a buffer, was due to the enzyme’s ability to catalyze the hydrolysis of phosphate bonds much more efficiently compared to its ability to catalyze the their hydrolysis in an aqueous buffer, which occurs slowly and spontaneously. Furthermore, due to hydrolytic degradation, the number of -P-O^−^groups increased, and they de-screened the attack of the negatively charged hydroxyl groups, which in this case acted as the hydrolyzing agent. The optimal pH conditions for ALP further enhanced this catalytic process, resulting in a significant increase in the degradation rate of the gel. This result confirmed that the PPAGHGel is biodegradable and a suitable candidate for biomedical applications, e.g., tissue engineering, or as a drug carrier.

### 2.5. Multivalent Studies of Polyphosphoramidate Glycohydrogel

One of the most important characteristics of glycohydrogels is their ability to recognize biomolecules and bind to them. The protein Con A has often been used as a crosslinking agent and is often immobilized in hydrogels to obtain glucose-sensitive gels. In the present work, an alternative approach was employed to obtain a glucose-containing gel that can recognize and bind to proteins. In the experiment, a solution of Con A at different concentrations in 0.1 M Tris-HCl buffer and at pH 7.5, containing 0.5 mM divalent metal ions (Mn^2+^), was used. The multivalent properties were proven by absorption analysis at a wavelength of 280 nm on a UV-VIS spectrometer.

**Figure 10:**
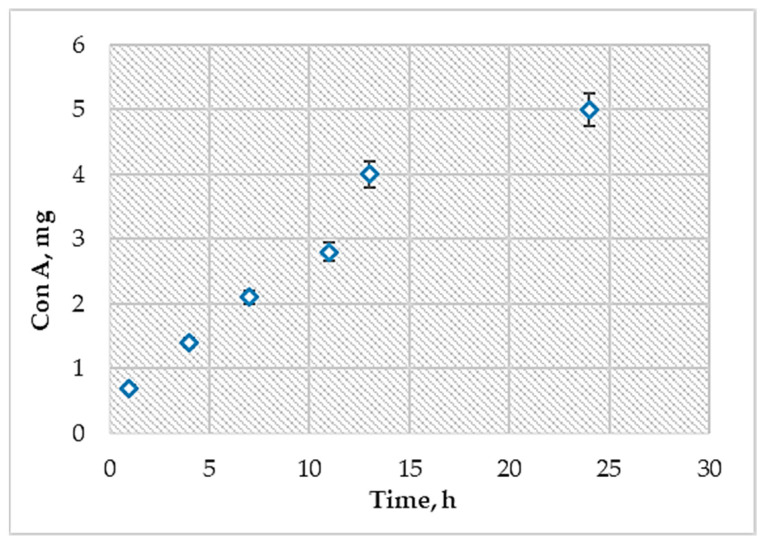
Content of Con A (mg) in PPAGHGel (m = 0.018 g) in TRIS-HCL buffer at pH = 7.5.

Using UV spectroscopy, it was found that PPAGHGel absorbs 92% of the maximum amount of protein with which it can complex within 24 h (Figure 10).

Solutions of glucose at concentrations of 0.02, 0.04, 0.06, 0.08, and 0.1 mM were added to the PPAGHGel. UV spectroscopy measurements revealed no leakage of the protein from the PPAGHGel, as no absorbance was detected at any of the concentrations tested. Even when the maximum glucose concentration of 0.12 mM, which corresponds to the highest amount the protein can bind, was added, the PPAGHGel–Con A complex remained intact (with absorbance at 280 nm still at zero). At a glucose concentration of 0.3 mM, three times the amount with which the protein can bind, the gel–protein complex remained stable, with no absorbance detected. However, at a glucose concentration of 0.32 mM, an absorbance of 0.1546 was observed, indicating that the complex began to disintegrate.

### 2.6. Preparation of Silver Nanoparticles

The presence of sugar structures in the gel allows for its use as a stabilizer for the preparation of silver nanoparticles, which have a strong antibacterial effect. Metal nanoparticles (silver, gold or copper) have antibacterial properties, which is why glycohydrogels with such nanoparticles show increased efficiency in the treatment of bacterial infections caused by *Staphylococcus aureus*, *Escherichia coli* or *Pseudomonas aeruginosa* [10].

In the polymer chain of PPAGHGel, glucose residues containing reactive –OH–groups are present, which turn the gel into a suitable material for the preparation of silver nanoparticles. Van der Waals interactions occur between the silver ions from silver nitrate and the –OH groups from the sugar residues in PPAGHGel. Then, the silver ions are reduced by glucose to obtain silver nanoparticles.

**Figure 11:**
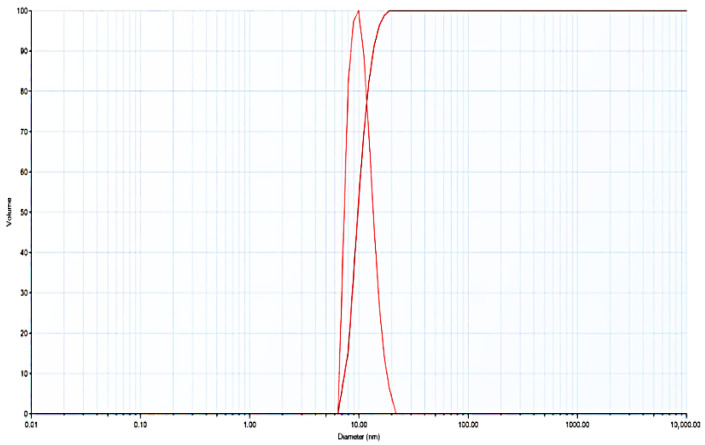
DLS–size distribution curve of Ag nanoparticles by volume at 20 °C.

The obtained silver nanoparticles were investigated by dynamic light scattering. Figure 11 presents the volume size distribution curve of the silver nanoparticles. In the figure, a monomodal distribution of uniform particles with an average hydrodynamic diameter (D_h_) of 12 ± 2 nm is observed. Martı’nez-Castan et al. obtained silver nanoparticles of different sizes (7, 29, 89 nm) [11]. The authors reported that the 7 nm silver nanoparticles showed the best antibacterial properties against *E. coli* and *S. aureus*, which are Gram-negative and Gram-positive bacteria, respectively. Due to their small size, 7 nm silver nanoparticles can easily reach the nucleus of bacteria, i.e., contact with bacteria is the greatest for silver nanoparticles of this size [12]. Therefore, the obtained silver nanoparticles, measuring 12.11 ± 1.93 nm, were assumed to possess strong antibacterial properties, and the PPAGHGel matrix was assumed as a suitable platform for their preparation.

### 2.7. Antibacterial Tests

Gels containing silver metal nanoparticles could be used as biomaterials with antibacterial properties. The antibacterial activity of the newly developed glycohydrogel was assessed by measuring the diameter of the sterile inhibition zones against *P. aeruginosa* and *S. aureus* (See Figure S1). The hydrogel containing silver nanoparticles (PPAGHGel-Ag) showed significant antibacterial activity against *P. aeruginosa*, with a sterile inhibition zone of 22.3 ± 0.4 mm (Table 1). Against *S. aureus*, the gel also demonstrated effective inhibition (17.5 ± 0.4 mm). The control probe, which was PPAGHGel without Ag nanoparticles, showed balanced activity against both microorganisms. These results suggest that PPAGHGel-Ag has strong antibacterial properties, especially against *P. aeruginosa*, and that modifying the hydrogel matrix boosts the material’s antibacterial effectiveness. This pronounced effect likely stems from the release of silver ions and their diffusion, i.e., PPAGHGel-Ag is a suitable candidate for the treatment of bacterial infections. It was shown in recent studies [13] that the antibacterial efficacy of hydrogels containing silver nanoparticles depends on many factors—the size of the nanoparticles, their release rate and their interaction with the bacterial surface. Therefore, our future work will focus on quantifying silver ion release and on further *in vitro* antibacterial tests to complement and deepen the promising results observed.

### 2.8. Cytotoxicity Studies

One of the essential requirements for biomedical applications of glycohydrogels is that they are non-toxic. In the present study, cytotoxicity was evaluated using the human cervical cancer cell line HeLa, a widely utilized model in biomedical research [14].

Cell viability was assessed using the MTT assay, which quantifies mitochondrial metabolic activity as a proxy for cell survival. Cells were incubated with various concentrations of PPAGHGel for 72 h, and the resulting data were used to construct dose–response curves and calculate the IC_50_ (the concentration at which cell viability is reduced by 50%).

**Figure 12:**
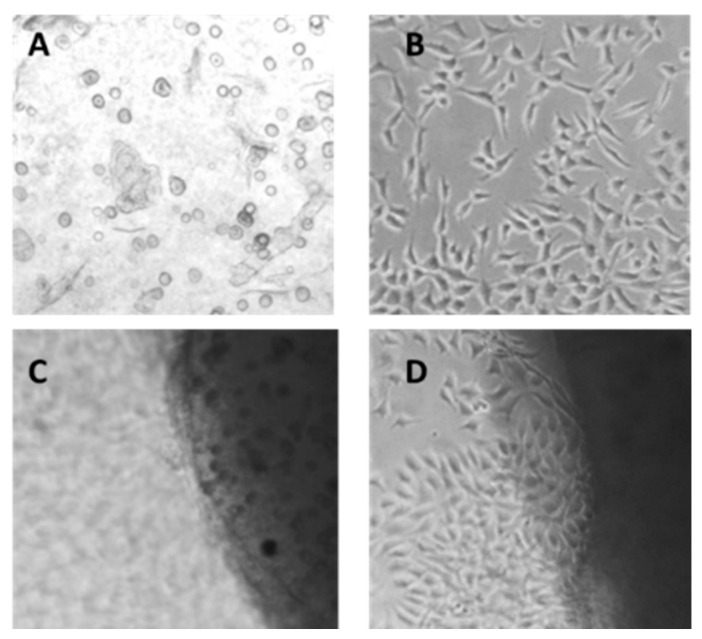
Microscopic observations of (**A**) the hydrogel; (**B**) *HeLa cells alone*; (**C**) cells co-cultured with the hydrogel (focus on the hydrogel); (**D**) cells co-cultured with the hydrogel (focus on the cells).

To investigate cellular interactions with PPAGHGel, we also assessed its bioadhesive properties. It is well known that when cells grow on different polymeric substrates, including glycohydrogels, their morphology and behavior may change [15]. Microscopy images (Figure 12) indicate that HeLa cells do not adhere to the surface of the gel. Importantly, there were no signs of cellular stress or death, such as rounding, vacuolization, or membrane blebbing.

**Figure 13:**
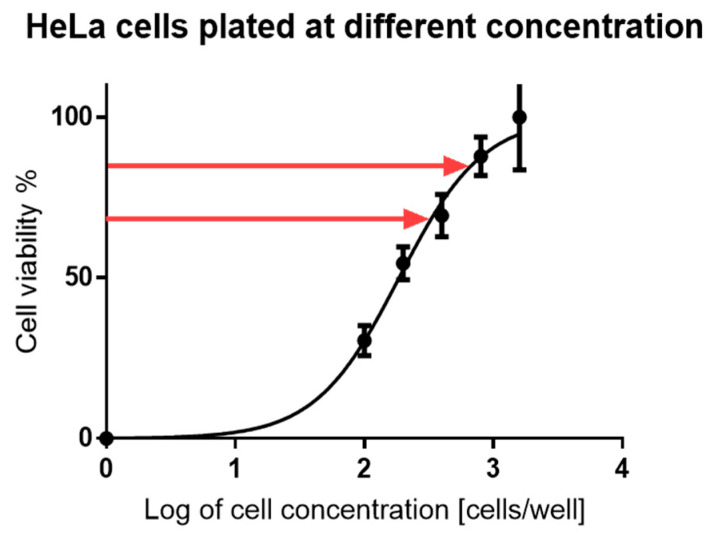
MTT assay results for HeLa cells cultured in the presence of PPAGHGel.

Cytotoxicity was further analyzed using a calibration curve constructed with serially diluted cell concentrations (1600, 800, 400, 200, and 100 µM cells/well). HeLa cells seeded on PPAGHGel disks at 800 µM and 400 µM maintained over 98% viability compared to untreated controls (Figure 13). These results confirm that PPAGHGel is non-toxic and does not impair cellular proliferation. However, the cells do not adhere to or invade the hydrogel matrix.

Taken together, these findings suggest that while PPAGHGel is not suitable for tissue engineering applications requiring cell attachment and integration, its excellent biocompatibility and non-adhesive properties make it a promising candidate for drug delivery systems or wound dressings.

## 3. Materials and Methods

### 3.1. Materials

All reagents, starting materials, and solvents were purchased from commercial suppliers. Reactions sensitive to moisture or air were performed in an argon atmosphere. Dimethyl H-phosphonate was distilled prior to use. Polyethylene glycol with a molar mass of 600 g mol^−1^ (PEG600) was purchased from Sigma-Aldrich ( Saint Louis, MO, USA) and dried by azeotropic distillation with toluene. Acetonitrile (MeCN) was dried over calcium hydride and distilled prior to use. Anhydrous dichloromethane (DCM), N,O-bis-trimethylsilyl acetamide (BSA) (≥99 wt%), tetrabutyl ammonium fluoride (TBAF) (≥32 wt%), silver nitrate (≥99 wt%) and hexamethyldisilazane (HMDS) (≥98 wt%) were purchased from Sigma-Aldrich. Tris (2,3-epoxypropyl isocyanurate), p-aminobenzoic acid (≥99 wt%), and Hexamethylene diisocyanate (≥99 wt%) (HMDI) were purchased from Fluka (Fluka Chemie GmbH, Buchs, Switzerland). Sodium azide (≥99 wt%) and Hexamethyldisilazane (HMDS) (≥98 wt%) were purchased from Merck (Merck, Darmstadt, Germany). Concanavalin A (Con A) (≥98 wt%) and D-glucosamine hydrochloride (≥98 wt%) were purchased from Carbosynth (Carbosynth Limited, Berkshire, UK).

### 3.2. Synthetic Procedures

#### 3.2.1. Preparation of Poly(oxyethylene H-phosphonate) (POEHP)

The synthesis of poly(oxyethylene H-phosphonate) (POEHP) was previously reported [16]. The synthetic procedure and product characterization are presented in the Supplementary Materials.

#### 3.2.2. Synthesis of Sugar Azide

Sugar azide was prepared in two steps. First, 2-amino-2-deoxy 1,3,4,6-tetra-O-trimethylsilyl-α-D-glucopyranose was obtained by applying a known procedure [17]. Next, 2-p-azidobenzamide-2-deoxy-1,3,4,6-tetra-O-trimethylsilyl-α-D-glucopyranose (AG) was synthesized following reported procedures [18]. These synthetic procedures are given in the Supplementary Materials.

#### 3.2.3. Synthesis of Poly(phosphoramidate) Glycoconjugate by Staudinger Reaction

PPAG was synthesized in three steps: first, it was silylated with a silylating agent, BSA, to obtain a tri-coordinated phosphorous polymer; the second step was the Staudinger reaction between the silylated polymer and azido-containing sugar (AG); finally, the product was desilylated using TBAF to obtain the desired phosphoramidate. The resulting polyphosphoramidate glycoconjugate was purified by dialysis. After dialysis, the product was lyophilized. The synthetic procedures are given in the Supplementary Materials.

#### 3.2.4. Synthesis of Phosphorus-Containing Polyphosphoramidate Glycohydrogel (PPAGHGel)

Poly(phosphoramidate) with 2-p-azidobenzamide-2-deoxy- 1,3,4,6-tetra-O-trimethylsilyl-α-D-glucopyranose (AG) (0.814 g, 1.26 mmol) was dissolved in dimethylformamide (DMF) (2 mL). Hexamethylene diisocyanate (HMDI) (0.12 g, 0.63 mmol) was also dissolved in DMF. The two solutions were stirred separately until they became completely transparent. After mixing them, the resulting mixture was stirred for 5 min and then placed in a desiccator containing dry silica gel for a period of 2 days to remove the solvent DMF. Then the mixture was heated at 60 °C for 30 min and then subjected to cooling at 12 °C for 1 h to yield a gelled material [19].

#### 3.2.5. Preparation of Silver Nanoparticles Using PPAGHGel

PPAGHGel was used in the following ways: (1) as a glycohydrogel containing silver nanoparticles, which have a strong antibacterial effect; (2) as a stabilizer for the preparation of silver nanoparticles. Silver nanoparticles in PPAGHGel were formed in several stages. The PPAGHGel network plays a role as a reducer of silver ions, since it contains glucose, which is a reducing agent. To produce these silver nanoparticles in the gel, it was diffused in 15 mL of an aqueous solution of silver nitrate at a concentration of 10 mM for 12 h in the dark. At this stage, most of the silver ions diffused into the network (pores) of the PPAGHGel and became bound to the –OH groups of the sugar residues along the polymer chain. The remaining silver ions occupies the free pores in the PPAGHGel. The resulting PPAGHGel containing silver nanoparticles was washed twice with deionized water to remove unbound silver particles and then immersed in 15 mL of a solution containing glucose (10 mM) and sodium hydroxide (10 mM and 50 mM) to induce the formation of silver nanoparticles. After 12 h, the gel solution was centrifuged for 2 min at 9000 rpm. The supernatant was decanted and examined using DLS.

### 3.3. Methods

#### 3.3.1. NMR and IR

^1^H, ^13^C, and ^31^P NMR spectra were recorded on Bruker 250 MHz and 600 MHz instruments (Billerica, MA, USA) in deuterated solvents, with the type of solvent indicated for each specific measurement.

#### 3.3.2. Sol–Gel Fraction

The sol–gel fraction of the PAGHGel was determined as follows: after gelation the gels were lyophilized and their weight was measured (M); then, the gels were stored in a mixture of EtOH/dest. H_2_O (20/80 vol%) for 48 h, and the solution was changed 10 times; finally, the samples were lyophilized again, and their final weight was determined (M′). The sol–gel fraction was calculated using the following formula:

(1) $$Gel\;fracition=\frac{M^{\prime }}{M}\times 100\%$$

#### 3.3.3. Swelling Degree

The swelling degree of the obtained gels was determined as following: the samples were lyophilized and their weight was measured (*m_j_*); after that, they were stored in a mixture of EtOH/dest. H_2_O (20/80 vol%) until no further change in the weight was noticed (until they were fully swollen) (*m_i_*). The swelling was calculated as follows:

(2) $$\%\;swelling=\frac{m_{i}-m_{j}}{m_{j}}\times 100\%$$

#### 3.3.4. Dynamic Light Scattering

Dynamic light scattering (DLS) measurements were performed with a NanoBrook 90Plus PALS Analyzer (Brookhaven instruments) and a Brookhaven Instrument Ltd. system equipped with a BI-200M goniometer (Brookhaven Instruments Corporation, Holtsville, NY, USA), a BI-APD dynamic correlator, BI-APD photomultiplier lamps and Melles Griot He-Ne lasers operating at a wavelength of λ = 632.8 nm. DLS measures Brownian motion and hydrodynamic particle size. The ζ-potential values were determined using the Smoluchowski equation relating ion mobilities to surface charge and were presented as the averages of five measurements. The dynamic light scattering apparatus consisted of 6 main components: 1. a laser—the source of sample illumination; 2. a cell—the place where the sample was placed; 3. a detector, which had 90° and 170° positions; 4. an attenuator, which controlled the intensity of the laser source; 5. a correlator, which transmitted the scattering intensity signal from the detector to the digital processor; 6. a computer for software analysis of the size data.

#### 3.3.5. Scanning Electron Microscopy (SEM)

A scanning electron microscope was used to determine the structure, phase composition and surface morphology of the PPAGHGel. The studies were carried out with the SEM Philips 515 (Philips Electron Optics B.V., Eindhoven, The Netherlands), a digitized scanning electron microscope with secondary electron imaging (SEI) detectors, an accelerating voltage of 30 kV, and a magnification of 40,000×. The sample was pre-metallized using Mini Sputter Coater SC7620 (EMS, Hatfield, PA, USA), which is a vacuum installation for sputtering carbon and noble metals and treating surfaces with a glow discharge. The hydrogel (PPAGHGel) was pre-swollen completely in deionized water at 25 °C and then lyophilized at −43 °C under a vacuum of 1 Pa for 24 h to avoid the destruction of the porous structure.

#### 3.3.6. Dynamic–Rheological Measurements

Dynamic–rheological measurements of the hydrogels were performed on a Haake RheoStress 600 rheometer (Thermo Fisher Scientific, Waltham, MA, USA) with a parallel plate sensor system (20 mm diameter) and a Peltier temperature controller. Disks of the gels were first extracted for 6 days at pH 7. The elastic modulus G′ and the deformation modulus G″ were measured in the frequency range 0.03–10 Hz at 25 °C in CD mode at γ = 0.005, which is included in linear viscoelastic mode. Three measurements were performed for each sample.

#### 3.3.7. Thermogravimetric Analysis

Thermogravimetric analysis was performed on a Perkin Elmer thermogravimetric analyzer (TGA 4000 System, 100–240 V/50–60 Hz) (PerkinElmer, Shelton, CT, USA). The glycohydrogel was initially lyophilized and then heated from room temperature to 260 °C at a rate of 10 °C/min in an argon atmosphere.

#### 3.3.8. Turbidimetry

Multivalent studies of PPAGHGel were performed with the protein Conanavalin A. According to theoretical calculations, a PPAGHGel with a mass of m = 0.018 g has the ability to react with a maximum amount of the protein Con A of m = 5 mg. The experiment used a solution of Con A (5 mg/mL) in Tris-HCl buffer at pH 7.5, containing 0.1 M NaCl, 0.5 mM CaCl_2_ and 0.5 mM MnCl_2_. The first step was to drop the protein solution onto the PPAGHGel (0.02 mL every 15 min). After each drop of the solution of Con A, the complexation between Con A and PPAGHGel was investigated, the process being monitored via absorption analysis on a UV-VIS spectrometer at a wavelength of 280 nm, the wavelength at which Con A absorbs. The decrease in the protein content in the solution was recorded; this decrease meant that the protein was absorbed by the gel and that there was possible complex formation. In order to prove that this decrease is indeed due to complex formation, and not swelling, an experiment was conducted to determine the stability of the complex. The maximum amount of glucose that could destroy the complex is c = 0.12 mM. The next step was to prepare glucose solutions of various concentrations: 0.02, 0.04, 0.06, 0.08 and 0.1 mM. Then, the solutions were added one by one to the complexed PPAGHGel–Con A. After each addition, the absorption was measured by a UV-VIS spectrometer at a wavelength of 280 nm, the wavelength at which glucose absorbs light.

#### 3.3.9. Purification Methods

PPAG was purified by dialysis in Spectra/Por Biotech CE membrane tubes (MWCO 3500) with distilled water at 2 °C for 48 h, with the dialysis water being changed every 2 h.

#### 3.3.10. Enzymatic and Hydrolytic Degradation

Enzymatic degradation of the PPAGHGel was performed with an alkaline phosphatase. The PPAGHGel was stored in Tris-HCl buffer with a pH of 9.0 at 37 °C containing ALP at a concentration of 500 U/L. The weight change in the gel and the absorbance at 600 nm on the UV-VIS spectrometer DU 800 (Beckman Coulter, Brea, CA, USA) were monitored.

#### 3.3.11. Antibacterial Tests

The antibacterial activity of the newly developed gel was assessed by examining its antagonistic effect against skin-associated pathogenic bacteria. Reference strains *P. aeruginosa* ATCC 9027 and *S. aureus* ATCC 6538 were used as test microorganisms. The bacterial cultures were grown in Luria–Bertani (LB) broth for 24 h at 37 °C, and then diluted with sterile physiological saline solution (0.9% NaCl) to a final concentration of 10^6^ colony-forming units per milliliter (CFU/mL). The overnight cultures were mixed with LB agar, cooled to approximately 45 °C, and poured into sterile Petri dishes. The PPAGHGel-Ag samples and the PPAGHGel, as the control material, each measuring approximately 1 cm^2^, were placed onto the solidified agar surface. The plates were kept at 4 °C for 1 h to promote nanoparticle diffusion, followed by incubation at 37 °C for 24 h. Antibacterial activity was determined by measuring the diameter of the inhibition zones (sterile halos) around the test samples, expressed in millimeters.

#### 3.3.12. Cytotoxicity Studies

The cytotoxicity of PPAGHGel was assessed using the MTT assay, following established protocols [20,21]. Briefly, HeLa cells were seeded at a density of 1 × 10^4^ cells per well in 96-well flat-bottom plates in 100 µL of complete growth medium. After 24 h of incubation at 37 °C in a humidified atmosphere with 5% CO_2_ to allow for cell attachment, the medium was replaced with 100 µL of fresh medium containing serial dilutions of PPAGHGel. Test concentrations ranged from 100 to 1600 µM, calculated based on the molar mass of the monomeric glucopyranose-containing units. Each concentration was tested in quadruplicate (*n* = 4). Cells were incubated with the test compounds for 72 h. Subsequently, 10 µL of MTT solution (final concentration 5 mg/mL) was added to each well, followed by 4 h of incubation at 37 °C. The resulting formazan crystals were solubilized with 100 µL of DMSO. Absorbance was measured at 550 nm with a reference wavelength of 630 nm using a Varioskan Lux spectrophotometer (Thermo Scientific, Waltham, MA, USA). Cell viability was expressed as a percentage relative to the untreated control cells. All experiments were independently performed at least three times (*n* = 3). Data were analyzed for statistical significance, with *p*-values < 0.05 being considered significant. The assay results confirmed the non-cytotoxic nature of PPAGHGel on HeLa cells across the tested concentration range.

#### 3.3.13. Cell Adhesion

For the cell adhesion assays, the HeLa cells were seeded directly onto pre-sterilized and pre-hydrated PPAGHGel disks (5 mm diameter) placed in standard 96-well culture plates. Adhesion and morphology were monitored by phase contrast microscopy after 24 and 72 h. Microscopic observations of cell adhesion on PPAGHGel were carried out using a Zeiss Axiovert 200M inverted microscope equipped with an AxioCam MRm CCD camera. Image acquisition was performed using AxioVision software (version 4.8.2). Primary image capture and processing were conducted using the native software, while secondary image analysis and processing were performed using ImageJ (version 2.1.0/1.53c, NIH, Bethesda, MD, USA). This setup enabled detailed visualization of cell morphology and interactions with the hydrogel surface.

## 4. Conclusions

For the first time, a polyphosphoramidate glycohydrogel (PPAGHGel) was obtained by crosslinking polyphosphoramidate glycoconjugate (PPAG) using hexamethylene diisocyanate (HMDI) as the crosslinking agent. Its isocyanate groups reacted with the hydroxyl groups of the glucose residues in the PPAG structure under mild reaction conditions. Successful preparation of PPAGHGel with a gel fraction of 83%, a swelling degree of 1426 ± 98% and good mechanical properties (G″ = 1560 ± 65 Pa) was demonstrated. Alkaline phosphatase (c = 400 U/L, pH = 9) completely degraded the gel in 19 days. For the same period, its hydrolytic degradation was 52% at pH = 9. The newly synthesized PPAGHGel was completely non-toxic. Multivalent studies confirmed the interaction between the synthesized PPAGHGel and Concanavalin A. Using UV spectroscopy, it was found that PPAGHGel absorbs 92% of the maximum amount of protein with which the gel complexes within 24 h. It was shown that there was no leakage of the protein from PPAGHGel. At a glucose concentration exceeding three times the amount which the protein can bind, the gel–protein complex remained stable. Upon further increases in the amount of glucose, the complex began to disintegrate. The morphology of PPAGHGel was studied by scanning electron microscopy. The pores on the surface of the gel had an average size of 10 µm. The presence of capillary channels meant that water was able to more easily access the interior of the gel, since the measured pore size was 0.1 mm. PPAGHGel is a suitable stabilizer for obtaining silver nanoparticles measuring 12 nm. PPAGHGel loaded with silver nanoparticles demonstrated antibacterial activity against Gram-negative and Gram-positive bacteria. The cytotoxicity evaluations showed that PPAGHGel is a biodegradable and non-toxic gel. In conclusion, besides being a suitable matrix for obtaining silver nanoparticles, the newly synthesized glycohydrogel is a promising material for different biomedical applications: biosensors, drug carriers or wound dressings.

## Figures and Tables

**Table 1:** Antibacterial activity of the new hydrogels against Gram-negative and Gram-positive microorganisms. Experiments were performed in triplicate.

Gel Type	Antibacterial Activity (Sterile Halo in mm)	
-	*P. aeruginosa*	*S. aureus*
PPAGHGel-Ag	22.3 ± 0.4 *	17.5 ± 0.4
PPAGHGel	20.3 ± 0.5	18.2 ± 0.4

* In addition to a sterile inhibition zone of 22.3 ± 0.4 mm, an additional bacteriostatic zone beyond 30 ± 0.4 mm was observed.

## Data Availability

The data are contained within this article and its Supplementary Materials.

## Supplementary Materials

The following supporting information can be downloaded at: https://www.mdpi.com/article/10.3390/molecules30153140/s1, Preparation of poly(oxyethylene H-phosphonate) (POEHP); Synthesis of p-azidobenzoic acid; Preparation of chloroazidobenzoic acid; Synthesis of amino sugar: (2-amino-2deoxy-1,3,4,6-tetra-O-trimethylsilyl-alpha-β-D-glucopyranose); Synthesis of azidosugar: (2-p-azidobenzamide-2-deoxy-1,3,4,6-tetra-O-trimethylsilyl-alpha--D-glucopyranose); Synthesis of poly(phosphoramidate)glycoconjugate (PPAG) by Staudinger reaction; Determination of molecular mass characteristics of POEHP and PPAG; Scheme S1: Synthesis of poly(oxyethylene H-phosphonate) (POEHP); Scheme S2: Synthesis of poly(phosphoramidate)glycoconjugate) (PPAG); Figure S1: Evaluation of the antibacterial activity of PPAGHGel-Ag against skin pathogenic bacteria. (a) inhibition of Pseudomonas aeruginosa growth (Gram-negative microorganism); (b) inhibition of Staphylococcus aureus growth (Gram-positive microorganism). Clear inhibition zones are visible around the applied hydrogels, indicating the extent of their antimicrobial activity. A bacteriostatic zone is visible around the gel against *P. aeruginosa*.; Figure S2: IR spectrum of POTMHP.

## Abbreviations

The following abbreviations are used in this manuscript:

PPAG	Polyphosphoramidate glycoconjugate
BSA	N,O-bis-trimethylsilyl acetamide
POEHP	Poly(oxyethylene H-phosphonate)
PPAGHG	Polyphopsphoramidate glycohydrogel
HMDS	Hexamethyldisilazane
TBAF	Tetrabutylammonium fluoride
PEG	Polyethylene glycol
HMDI	Hexamethylene diisocyanate
ALP	Alkaline phosphatase
PVA	Polyvinyl alcohol
AG	Amino-containing glucose
