# Supplementary material for: Polyphosphoramidate Glycohydrogels with Biorecognition Properties and Potential Antibacterial Activity
Source: Molecules. 2025 Jul 26;30(15):3140. doi: 10.3390/molecules30153140 (PMC12348332; doi:10.3390/molecules30153140)
Supplement: Supplementary file 1 [file molecules-30-03140-s001.zip › molecules-3731333-supplementary.pdf]

Supplementary Materials

Polyphosphoramidate Glycohydrogels with Biorecognition Properties and Potential Antibacterial Activity

Zornica Todorova [1,2], Oyundari Tumurbaatar [1,2], Violeta Mitova [1,] Neli Koseva [1], Iva Ugrinova [3], Penka Petrova [4] and Kolio Troev [1,*]

1 Institute of Polymers, Bulgarian academy of Sciences; ztodorova@polymer.bas.bg
2 Institute of Organic Chemistry with Centre of Phytochemistry; Bulgarian Academy of Sciences; Zornica.Todorova@orgchm.bas.bg
3 Institute of Molecular Biology, Bulgarian Academy of Sciences; imb@bio21.bas.bg
4 The Stephan Angeloff Institute of Microbiology, Bulgarian Academy of Sciences; pepipetrova@yahoo.com
* Correspondence: ktroev@polymer.bas.bg

Preparation of poly(oxyethylene H-phosphonate) (POEHP)

Polyethylene glycol (PEG 600) (21.37 g, 35.61 mmol) and dimethyl H-phosphonate (4.70 g, 42.70 mmol)) were mixed in 20% stoichiometric excess and stirred in an Ar atmosphere with a magnetic stirrer in a round-bottomed flask equipped with a thermometer and a straight condenser. The by-product (methanol) was removed at normal pressure. The step was continued until the amount of methanol separated reached 80-90% of the theoretical yield. Then the temperature was slowly increased to 160 ° C and a vacuum (0.1 mbar) was applied. The temperature was maintained at 180 ° C for about 2 hours. The resulting poly(oxyethylene H-phosphonate) (POEHP) was highly viscous, colorless and upon cooling transformed into a wax-like product. It is soluble in water, ethanol, dichloromethane, chloroform and tetrahydrofuran. The polymer is insoluble in carbon tetrachloride, diethyl ether and hexane.

^{1}H NMR (600 MHz, $CDCl_3$) δ (ppm): 3.31-3.65 (m, OCH_2CH_2); 3.72 (d, ^{3}J (P,H) = 12 Hz, -$POCH_3$); 4.09-4.20 (m, P-OCH_2-); 6.81 (d, ^{1}J(P,H) = 711.55 Hz, -CH_2O-P(O)(H)(OCH_3) end group); 6.89 (d, ^{1}J(P,H) = 717.96 Hz, -CH_2O-P(O)(H)OCH_2- repeating unit), 6.76 (d, ^{1}J(P,H= 704.55 Hz, HO–P(O)(H)OCH_2CH_2O- end group.

^{31}P{H} (600 MHz, $CDCl_3$) δ (ppm): 9.32 (s, –CH_2O-P(O)(H)OCH_2-repeat unit; 10.01(s, CH_3O–P(O)(H)OCH_2CH_2O-), 7.29 (HO–P(O)(H)OCH_2CH_2O).

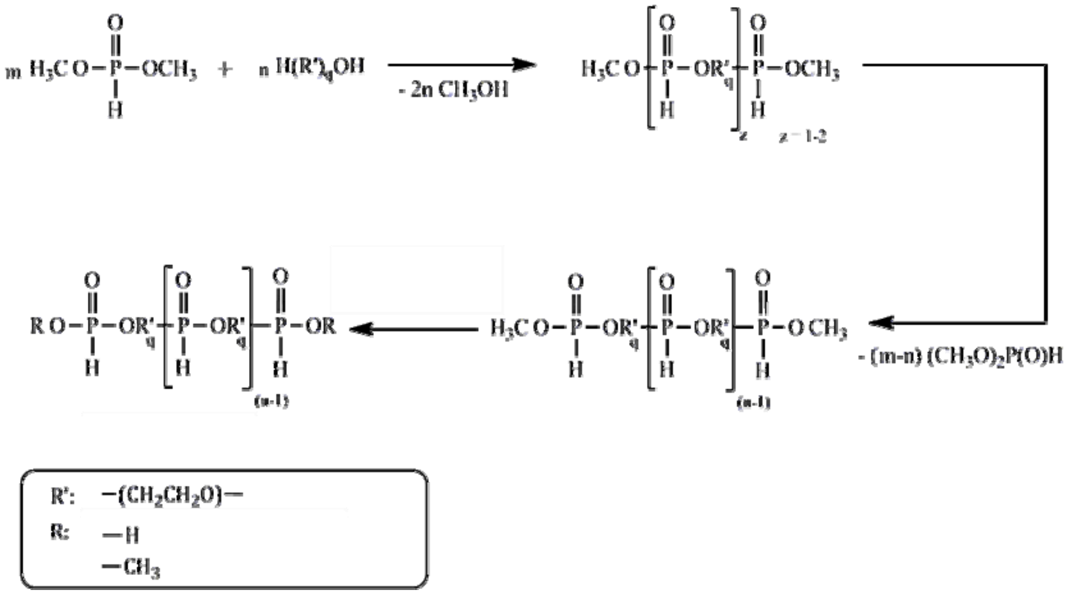

Scheme S1. Synthesis of poly(oxyethylene H-phosphonate) (POEHP)

Synthesis of *p*-azidobenzoic acid

4-Aminobenzoic acid (6.00 g, 43.75 mmol) was dissolved in 10 ml of deionized water and concentrated sulfuric acid (98%, 9 ml) and an additional 10 ml of deionized water was added. The solution was cooled to 0°C and a solution of $NaNO_2$ (3.18 g, 52.17 mmol) and water (9 ml) was added under constant stirring. Sodium azide (3.6 g, 55.38 mmol) was added until a brown solution was obtained. After an additional 10-15 min stirring at 0°C, the precipitate was filtered and washed several times with water. 4-Azidobenzoic acid was dissolved in ethyl acetate and dried with MgSO4. Yield: 75 %.

^{1}H NMR (250 MHz, CD_3OD): δ (ppm)= 8.17 – 7.89 (t, 2H, CH), 7.24 – 6.90 (t, 2H, CH), 4.84(s, 1H, OH).

Preparation of chloroazidobenzoic acid

p-Azidobenzoic acid (3.00 g, 18.3 mmol) was dissolved in 10 ml of thionyl chloride and 5 ml of dichloromethane in a round-bottomed flask equipped with a reflux condenser. The solution was heated to 75°C for 5 hours. Then the resulting mixture was washed 3 times with dichloromethane, which was removed with a rotary evaporator. The yield of the finished product was 70%.

^1H NMR (250 MHz, CD_3OD): δ (ppm)= 8.16 – 7.90 (t, 2H, CH), 7.30 – 6.85 (t, 2H, CH).

Synthesis of amino sugar: (2-amino-2deoxy-1,3,4,6-tetra-O-trimethylsilyl-alpha-β-D-glucopyranose)

To a suspension of D-glucosamine hydrochloride (2.00 g, 11.16 mmol) in acetonitrile (10 ml, dry) was added HMDS (4.91 ml 23.44 mmol) at room temperature under Ar atmosphere, and the mixture was allowed to stir at room temperature for 3 h. The mixture was then filtered and the filtrate was evaporated under reduced pressure to give the desired product as a colorless crystalline solid. Yield 97%.

^1H NMR (600 MHz, $CDCl_3$) δ: 5.07 (d, J=3.2 Hz, 1H), 3.61 (ddd, J=9.4, 4.3, 2.5 Hz, 1H), 3.50 (t, J=8.9 Hz, 1H), 3.43 (t, J=8.9, 1H), 2.50 (dd, 1H, J=9.0, 3.2 Hz), 3.71 – 3.66 (t, 2H), 0.17 (s, 9H, TMS), 0.14 (s, 9H, TMS), 0.13 (s, 9H, TMS), 0.06 (s, 9H, TMS).

^{13}C NMR (600 MHz, $CDCl_3$) δ: 94.5 (CH), 76.9 (CH), 73.0 (CH), 71.0 (CH), 63.7 (CH_2), 57.0 (CH), 1.5(CH_3), 1.0 (CH_3), 0.1 (CH_3), 0.04 (CH_3).

Synthesis of azidosugar: (2-p-azidobenzamide-2-deoxy-1,3,4,6-tetra-O-trimethylsilyl-alpha--D-glucopyranose) (Smith et al. 2003)

To a solution of 2-amino-2-deoxy-1,3,4,6-tetra-O-trimethylsilyl-alpha-β-D-glucopyranose (3.45 g, 7.37 mmol) in CH_2Cl_2:Pyr (24.15:10.35 ml) was added p-azidobenzoyl chloride (1.18 g, 8.1 mmol) at 0 °C and the mixture was stirred for 2 h. The mixture was extracted twice with 10% copper (CuSO4*5H2O) solution. After extraction, the color of the organic phase changed to green. Then the organic phase is collected and extracted again 2-3 times with 10% EDTA until the organic phase is yellow. The extract is evaporated under vacuum to obtain the desired product. Yield: 70%.

^1H NMR (600 MHz, DMSO) δ: 7.64 (d, J = 9.0 Hz, 1H); 6.97 (d, j = 8.75 Hz, 2H); 6.91 (d, J = 8.37 Hz, 2H); 5.93 (d, J= 9.56 Hz, 1H); 5.00 (t, j = 9.41 Hz, 1H); 4.06-4.15 (m, 2H); 3.51-3.59 (m, 2H), 1.86 (s, 3H).

Synthesis of poly(phosphoramidate)glycoconjugate (PPAG) by Staudinger reaction

POEHP (3.50 g, 5.42 mmol) was dissolved in dichloromethane and BCA (4.41 g, 21.67 mmol) was added to the reaction mixture at room temperature and under an inert atmosphere. After 1 h of stirring, a solution of azidosugar (2-p-azidobenzamide-2-deoxy-1,3,4,6-tetra-O-trimethylsilyl-alpha-β-D-glucopyranose) (3.32 g, 5.42 mmol) in the same solvent was added and the mixture was stirred for an additional 16 h. The product was then desilylated using tetrabutylammonium fluoride (TBAF) (11.33 g, 43.44 mmol) to obtain the desired phosphoramidate. The resulting polyphosphoramidate glycoconjugate was purified by dialysis. After dialysis, the product was lyophilized.

^{1}H NMR (600 MHz, DMSO) δ: 7.06, 7.77 (aromatic protons of azidosugars), 4.02-4.09 (-CH_2OCH_2-oxymethylene protons of POEHP), 3.14-3.64 (-CH_2OCH_2- oxymethylene protons of the repeating unit).

^{31}P{H} NMR (600 MHz, DMSO) δ: 2.34 (s, for phosphorus nucleus, bonded to nitrogen of the amido group in the repeating unit), 1.79 (s, for phosphorus nucleus, bonded to nitrogen of the amido group in the end group).

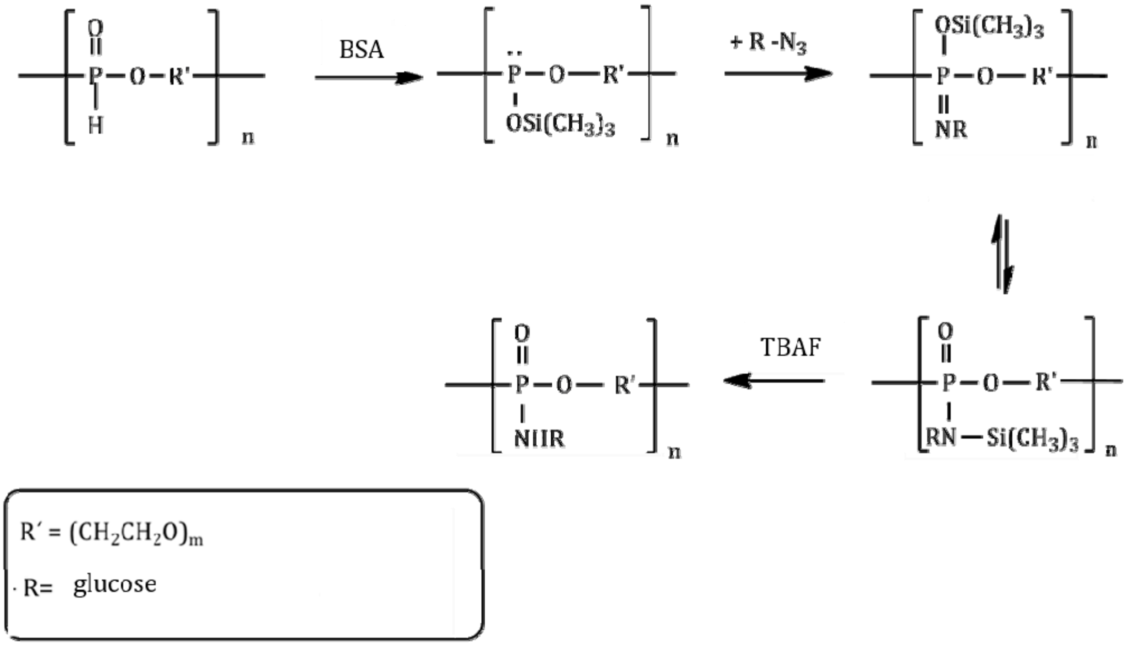

Scheme S2. Synthesis of poly(phosphoramidate)glycoconjugate) (PPAG)

Determination of molecular mass characteristics of POEHP and PPAG

The ratio of the integral intensity of the signal for the P-H protons in the repeating unit (A1) to that of the signal for the P-H-protons of the terminal units (A2 and A3) gives the degree of polymerization, which is used to calculate the average molecular weight of the polymer Mn. The number of the repeating unit of POEHP according to the ^{1}H NMR spectrum is 6, and according to the ^{31}P spectrum is also 6. It is calculated by the following formulas:

$$\mathrm{n} = \frac{A_1}{(A_2 + A_3)/2}, \tag{S1}$$

$$M_n = n \times \mathrm{M}_{repeating\ unit} + \mathrm{M}_{\mathrm{end\ group\ 1}} + \mathrm{M}_{\mathrm{end\ group\ 2}} \tag{S2}$$

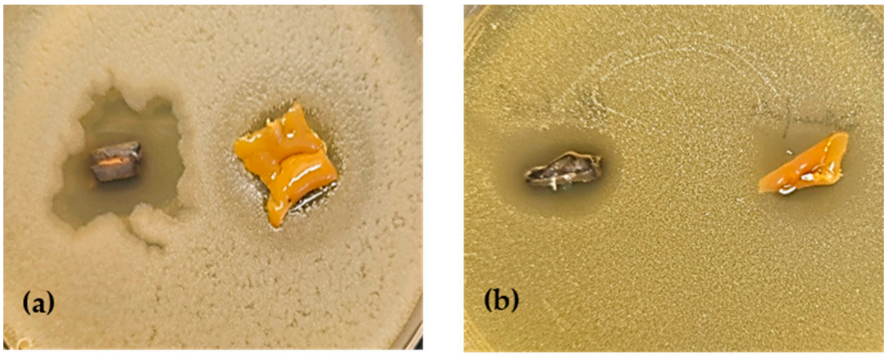

Figure S1. Evaluation of the antibacterial activity of PPAGHGel-Ag against skin pathogenic bacteria. a) inhibition of *Pseudomonas aeruginosa* growth (Gram-negative microorganism); b) inhibition of *Staphylococcus aureus* growth (Gram-positive microorganism). Clear inhibition zones are visible around the applied hydrogels, indicating the extent of their antimicrobial activity. A bacteriostatic zone is visible around the gel against *P. aeruginosa*.

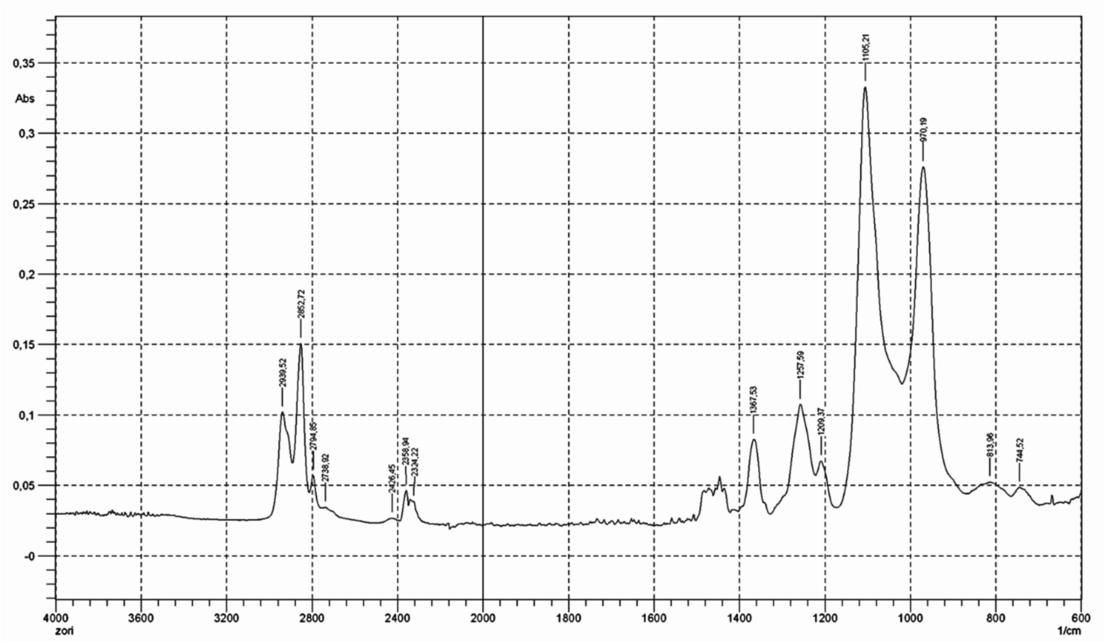

Figure S2. IR spectrum of POTMHP.

The infrared spectra of POTMHP present the characteristic absorption bands: at 2440 cm^{-1} for phosphonate groups (ν(P-H)), in the region1252-1257 cm^{-1} for the phosphoryl groups (ν(P=O)), as well as an intensive absorption band in the region of 1160-1000 cm^{-1} due to valent oscillations of the P-O-C bonds (ν($POCH_2$)) and the ether linkages (ν(CH_2-O-CH_2)) from the units. The absorption of the methylene groups is in the characteristic areas of 3000-2750 cm^{-1} and 1470-1340 cm^{-1}, respectively for valent (ν (C-H)) and deformation (δ (C-H)) oscillations.
